# S100A7/psoriasin expression in the human lung: unchanged in patients with COPD, but upregulated upon positive *S. aureus *detection

**DOI:** 10.1186/1471-2466-11-10

**Published:** 2011-02-15

**Authors:** Ellen Andresen, Christoph Lange, Daniela Strodthoff, Torsten Goldmann, Nicole Fischer, Hany Sahly, Detlev Branscheid, Holger Heine

**Affiliations:** 1 Division of Innate Immunity, Department of Immunology and Cell Biology, Research Center Borstel, Leibniz-Center for Medicine and Biosciences, Parkallee 1-40, 23845 Borstel, Germany; 2 Division Clinical Infection Diseases, Department of Pneumology, Research Center Borstel, Leibniz-Center for Medicine and Biosciences, Parkallee 1-40, 23845 Borstel, Germany; 3 Division of Clinical and Experimental Pathology, Department of Pneumology, Research Center Borstel, Leibniz-Center for Medicine and Biosciences, Parkallee 1-40, 23845 Borstel, Germany; 4 Institute for Medical Microbiology and Virology, University Medical Center Eppendorf, Martinistrasse 52, 20246 Hamburg, Germany; 5 IPM Institute for Immunology Clinical Pathology Molecular Medicine, Lademannbogen 61, 22339 Hamburg and Institute for Infection Medicine, University Medical Center Schleswig-Holstein Campus Kiel, Christian-Albrecht University of Kiel, Brunswiker Str. 4, 24105 Kiel, Germany; 6 Department of Thoracic Surgery, Grosshansdorf Hospital, Wöhrendamm 80, 22927 Großhansdorf, Germany

## Abstract

**Background:**

Progressive airway inflammation and susceptibility to the airway colonisation and infection are characteristic for the pathophysiology of chronic obstructive pulmonary disease (COPD). Antimicrobial peptides (AMPs) are central to the function of the innate host immune response against microbial pathogens and are regulators of inflammation and immunity. S100A7/psoriasin, a recently described AMP, is an essential component of the human epithelia against invading pathogens and acts as an effector molecule of the host innate defence in the skin. We hypothesized that S100A7/psoriasin is involved in the airway mucosal immunity and differently regulated and expressed in the lung during progression of COPD.

**Methods:**

S100A7/psoriasin gene expression was assessed in bronchial biopsies and bronchoalveolar lavage (BAL) fluid cells of healthy controls and COPD patients. Using confocal microscopy and immunohistochemistry, the protein expression of S100A7/psoriasin was investigated.

**Results:**

Here, we report that S100A7/psoriasin, the major antimicrobial peptide of the human skin, is constitutively expressed in perinuclear granules of human bronchial epithelial cells and alveolar macrophages. Whereas typical activators of the innate immune response like TLR ligands and cytokines induced the upregulation of CXCL-8 mRNA and release of CXCL-8 by epithelial cells, S100A7/psoriasin mRNA expression was not modulated. To investigate a potential association of S100A7/psoriasin with COPD, S100A7/psoriasin mRNA expression was assessed in bronchial biopsies and BAL fluid cells of patients at different stages of COPD and controls. Overall, 10 healthy individuals and 34 COPD patients were enrolled in this study. We found an association of S100A7/psoriasin mRNA expression with bacterial detection in the tracheobronchial system (p = 0.0304), which was the strongest in individuals positive for with *S. aureus *(p = 0.0005). However, S100A7/psoriasin mRNA expression was not altered during the progression of COPD.

**Conclusions:**

S100A7/psoriasin gene expression is unchanged in the airways during COPD. The newly identified association of S100A7/psoriasin with *S. aureus *may provide new insights into the antimicrobial defence response of the human airways, leading to the induction of S100A7/psoriasin upon microbial challenge.

## Background

The human lung serves as the largest interface between the host and the environment, and its immune defenses are critical for survival of the individual. A healthy adult ventilates 16,000 - 24,000 times per day on average. As the inhaled air is not sterile, respiratory cells are frequently exposed to airborne microorganisms, including pathogenic bacteria, viruses and fungi. Thus, an effective innate immune system is required to protect the airways and lungs from infection. Polymorphonuclear granulocytes, alveolar macrophages and epithelial cells play an important role in the innate immune response of the lung [1]. Many of the receptors employed by the innate immune system to identify potential pathogens have been characterized [2,3]. The effector molecules secreted by epithelial cells include cytokines, chemotactic factors as well as a variety of antimicrobial substances [4,5]. Among these substances are C-type lectins such as SP-A and SP-D [6], LL37/CAP-18 [7], SLPI [8], and antimicrobial peptides such as β-defensins [9-12].

The role of antimicrobial peptides (AMPs) in the innate immune defense mechanisms of human airways and lungs has recently gained more clinical interest [13]. Among a group of recently described antimicrobial peptides that are produced by human epithelial cells, S100A7/psoriasin exhibits very strong antimicrobial activity at low molecular concentrations [14], which was confirmed by comparing the activity of natural N-acetylated S100A7/psoriasin with that of chemically synthesized N-acetylated S100A7 [15].

S100A7/psoriasin belongs to the S100 family of EF-hand calcium-binding proteins which consists of more than 20 members with very distinct functions [16], ranging from differentiation, cell cycle progression, intracellular Ca^2+ ^signaling to leukocyte chemotaxis [17,18].

Chronic obstructive pulmonary disease (COPD) is one of the leading causes of mortality and morbidity worldwide [19]. The clinical hallmark of COPD is chronic bronchitis and emphysema with progressive respiratory tract inflammation and recurrent exacerbations at advanced stages of the disease. Colonization of the respiratory tract with various microorganisms is frequently observed as COPD progresses and most exacerbations are attributed to bacteria or viruses [19]. However, little is known about specific components of innate immunity that play a role in COPD progression. We hypothesized that S100A7/psoriasin involved in the airway mucosal immunity is differently regulated and expressed in the lung during pathogenesis of COPD. Furthermore, an association of S100A7/psoriasin gene expression with positive microbial detection of the respiratory tract was investigated.

## Methods

### Ethics statement

This study was approved by the Ethic Committee of the Medical Faculty of the University of Lübeck. All study participants provided written informed consent for the collection of bronchial biopsies and BAL fluids.

### Patients

Healthy volunteer controls and individuals with stages 1-4 COPD were recruited at the Medical Clinic of the Research Center Borstel, Germany. We followed the guidelines for grading disease severity in COPD of the Deutsche Atemwegsliga and the Deutsche Gesellschaft für Pneumologie (DGP), which is based on the international GOLD classification and defined by an FEV_1_/VC < 70% and FEV_1 _< 80% of the predicted value [20,21]. Following written informed consent bronchoscopy was performed according to national guidelines [22] obtaining bronchial biopsies from the middle lobe carina and BAL fluid with 200-300 ml of normal saline.

BAL fluid and biopsy specimens were collected from ten healthy subjects, two patients with mild COPD (stage 1), 13 patients with moderate COPD (stage 2), 15 patients with severe COPD (stage 3) and four patients with very severe COPD (stage 4). Healthy subjects for bronchoscopy were volunteers with FEV_1_/VC > 70% of the predicted value who were recruited by advertisement on the basis that they did not have signs or symptoms of COPD. Chronic illnesses (e.g. diabetes mellitus, renal insufficiency, HIV-infection) were exclusion criteria for study participant. Body plethysmography was performed using Jaeger MasterScreen 601045. The 6-min walking test (6MWD), arterial blood gas analysis, assessment of the MRC dyspnoea scale and calculation of probability of survival based on BODE score [23] completed the clinical investigations. COPD exacerbations have been defined according to Anthonisen criteria [24]. The basic characteristics of the 34 COPD patients with stages 1-4 and 10 healthy controls are shown in Table 1.

**Table 1:** Baseline characteristics of the study participants providing BAL fluid and biopsy specimens

Characteristic	Healthy controls	COPD patients			
		
		Stage 1	Stage 2	Stage 3	Stage 4
Number	10	2	13	15	4

Age [years]	31.3 ± 7.8 (20-46)	73.5 ± 7.8 (68-79)	66.9 ± 9.9 (52-82)	66.4 ± 7.5 (46-77)	58.3 ± 3.6 (55-63)

Sex [M/F]	3/7	1/1	9/4	10/5	2/2

BMI	22.7 ± 1.9 (20.0-26.7)	27.2 ± 0.2 (27.0-27.3)	28.3-5.8 (22.5-44.6)	25.4 ± 4.7 (16.0-35.5)	21.3 ± 3.7 (16.2-24.5)

Pack years	2.9 ± 2.8 (1-7)	50.0 ± 14.1 (40-60)	41.9 ± 19.3 (10-85)	39.7 ± 16.6 (5-65)	42.5 ± 18.9 (30-70)

6MWD [m]	516.0 ± 49.3 (430-625)	305.0 ± 169.7 (185-425)	352.3 ± 127.1 (150-545)	291.0 ± 122.3 (0-465)	367.5 ± 88.6 (240-430)

FEV_1 _[%]	104.2 ± 16.6 (60.7-117.4)	89.9 ± 2.0 (88.5-91.3)	63.9 ± 10.2 (50.1-79.4)	38.2 ± 6.2 (30.2-47.3)	26.1 ± 2.8 (22.8-29.0)

VC [%]	108.5-26.4 (54.4-160.9)	107.3 ± 2.3 (105.7-108.9)	86.3 ± 12.4 (65.2-108.9)	69.2 ± 14.7 (42.6-97.7)	61.3 ± 10.6 (52.8-76.3)

FEV_1_/VC [%]	88.7 ± 8.6 (73.5-101.5)	73.1 ± 11.2 (65.2-81.0)	65.6 ± 12.6 (49.5-90.7)	46.8 ± 10.3 (30.0-66.3)	39.1 ± 7.5 (31.5-46.3)

BODE score	†	2.0 ± 2.8 (0.0-4.0)	2.2 ± 2.3 (0.0-6.0)	5.1 ± 2.1 (2.0-9.0)	6.3 ± 1.0 (5.0-7.0)

Data are presented as mean ± SD (range), COPD: chronic obstructive pulmonary disease, M: male, F: female, BMI: body mass index, pack years: the number of cigarettes smoked per day × number of years smoked)/20 (1 pack has 20 cigarettes), 6MWD: 6-minutes walking distance, FEV_1_: forced expiratory volume in one second, VC: vital capacity, BODE score: index, which incorporates body mass index, airflow obstruction, dyspnoea and exercise capacity [22],% of predicted value, † not determined. Stages 1 through 4 denote severity of disease according to the Deutsche Atemwegsliga and the Deutsche Gesellschaft für Pneumologie [20], with higher number indicating greater severity.

For the staining of peripheral lung tissue we obtained specimens of lung tissue from surgical specimens that were resected from patients (n = 3) with the diagnosis of bronchogenic carcinoma (informed consent obtained) and routinely diagnosed at the Division of Pneumology at the Research Center Borstel. The specimens used were tumor-free material at least 5 cm away from the tumor front and were fixed then embedded in paraffin using the HOPE (HEPES-glutamic acid buffer mediated organic solvent protection effect) technique [25].

### Detection of microbes in human material

Microbes were detected (BAL fluid, biopsy specimen) following standard laboratory procedures. Human material was inoculated on 5% sheep-blood agar, salt mannitol agar and Tarozzi bouillon for 48 h at 37°C. Subcultures of Tarozzi bouillon followed after 24 h incubation at 37°C to augment detection of bacteria. Pure monocultures of *E. coli *and *S. aureus *were subjected to biotyping using automated VITEK 2 biotyping system, the API20E and the ID 32 Staph system (bioMérieux, Marcy l'Etoile, France). *S. aureus *was additionally identified by their ability to coagulate citrate plasma and to cleave DNA and mannitol. Virochip microarrays used in this study were identical to those previously described [26]. RNA isolated from BAL fluid were amplified and labeled using a modified Round A/B random PCR method and hybridized to the Virochip microarrays as reported previously [26,27].

### BAL fluid and bronchial biopsies preparation

BAL fluid was centrifuged (at 500 × g for 10 min, 4°C) and the sedimented cells washed twice with PBS (at 500 × g for 5 min, 4°C). Cell viability was assessed with the use of the trypan-blue exclusion method and always above 95%. Bronchial biopsy specimens (two pieces from each study participant) were homogenized with the use of the Pellet Pestle Cordless Motor (Kontes, New Jersey, USA).

### Cell culture

NCI-H727 (bronchial epithelial cell line) and A549 (lung epithelial cell line) (both from ATCC, Manassas, VA) were cultured at 37°C and 5% CO_2 _in RPMI1640 and DMEM medium, respectively, supplemented with 10% fetal calf serum and 1% of penicillin/streptomycin. For treatments cells were cultured for the time indicated in the presence of 100 ng/ml LPS (from *Salmonella enterica*, serovar *Friedenau*, kindly provided by Prof. Dr. H. Brade, Research Center Borstel, Gemany), 50 U/ml IL-1β (Strathmann Biotech GmbH, Germany), or 5 ng/ml recombinant human TNF-α (kindly provided by Prof. Dr. D. Männel, Regensburg, Germany). For controls, only medium supplemented with 10% fetal calf serum and 1% of penicillin/streptomycin was added to the cells. S100A7/psoriasin and CXCL-8 mRNA expression was determined as well as CXCL-8 production using an ELISA (Biosource, Nivelles, Belgium) on cell culture supernatants.

### Real-time quantitative PCR

Total RNA was isolated from cells of BAL fluid, homogenized lung biopsy specimens and cultured lung epithelial cells with the Absolutely RNA kit (Stratagene, La Jolla, CA, USA). Reverse transcription was performed in the presence of Superscript III Reverse Transcriptase (Invitrogen, Carlsbad, CA, USA). Gene transcript levels of S100A7/psoriasin, CXCL-8, β_2 _microglobulin (β_2_-M) and porphobilinogen deaminase (PBG-D) were quantified by real-time PCR with the use of LightCycler 480 Probes Master, S100A7/psoriasin-specific primers and the universal probe #60 (for S100A7/psoriasin) or LightCycler 480 SYBR Green I Master (for CXCL-8, β_2_-M and PBG-D) on a LightCycler 480 Instrument (Roche Applied Science, Mannheim, Germany) according to the manufacturer's instructions. Standard curves were obtained for each primer set with serial dilutions of plasmid DNA containing the amplification product. Absolute transcript levels are shown per one transcript of β_2_-M or as a common logarithm of this ratio for BAL fluid cells and bronchial biopsies. For cell lines NCI-H727 and A549, variations in the amount in different samples were corrected by PBG-D expression. Sequences of the used primer sets were: S100A7/psoriasin: sense 5'-CTGCTGACGATGATGAAGGA-3', antisense 5'-CGAGGTAATTTGTGCCCTTT-3'; CXCL-8: sense 5'-TTGCCAAGGAGTGCTAAAGAA-3', antisense 5'-CAACCCTACAACAGACCCACAC-3'; β_2_-M: sense 5'-GCTGTGCTCGCGCTACTCTC-3', antisense 5'-GCGGCATCTTCAAACCTCCAT-3', PBG-D: sense 5'-AACCCTGCCAGAGAAGAGTG-3', antisense 5'-AGCCGGGTGTTGAGGTTT-3'.

### Cell staining

A549 cells were fixed with 2% paraformaldehyde in PBS for 10 min. Cells were permeabilized with 0.25% Triton X-100 in PBS for 5 min, incubated with the monoclonal antibody to psoriasin/HID5/S100A7 (1:200, clone 47C1068, isotype Mouse IgG1, κ, Imgenex, San Diego, CA, USA) or mouse IgG1 (1:200, BD Biosciences, Heidelberg, Germany), washed and incubated with Alexa Fluor 546 dye-conjugated goat-anti-mouse antibody (1:300, Molecular Probes, Eugene, USA). Cells were stained with TOTO-3-iodid (1:500, Invitrogen, Carlsbad, CA, USA) and examined using a Leica confocal laser scan microscope TCS SP1 (Bensheim, Germany). All antibodies were diluted in PBS containing 10% BSA and incubated for 30 min.

### Immunohisto- and Immunocytochemistry

Sections of 4 μm of HOPE-fixed, paraffin-embedded surgical lung specimens were cut, deparaffinized and stained with the psoriasin/HID5/S100A7 antibody (1:500 in PBS). Signals were detected by sequential incubation with biotinylated anti-mouse antibody and peroxidase-conjugated streptavidin reagent (both 3-fold diluted in PBS) by using 3-Amino9-Ethylcarbazole/H_2_O_2 _as chromogen (DAKO, Denmark). Counterstain was performed with Mayer's hemalum before visualization of the immunoreactions. NCI-H727 cells were fixed with HOPE technique [26] for 36 h and stained with the Psoriasin/HID5/S100A7 antibody as described above. Negative controls, where primary antibody was omitted, were included.

### Statistical analysis

All results are expressed as mean ± SD or mean ± SEM, whereby each symbol represents a single sample. The one-tailed hypothesis was tested using unpaired t-test from log transformed data. Non-normal distributed values were tested with Mann-Whitney test. Analysis of variance of three or more unmatched groups was performed with the use of the One-way ANOVA test followed by Tukey's Multiple Comparison test or nonparametric Kruskal-Wallis test. When the results were significant, the unpaired t-test or Mann-Whitney test, respectively, was performed for comparison between the groups. Statistics were performed with GraphPad Prism 5.02 software (GraphPad, San Diego, CA, USA) where differences with p < 0.05 were considered significant.

## Results

**Figure 1:**
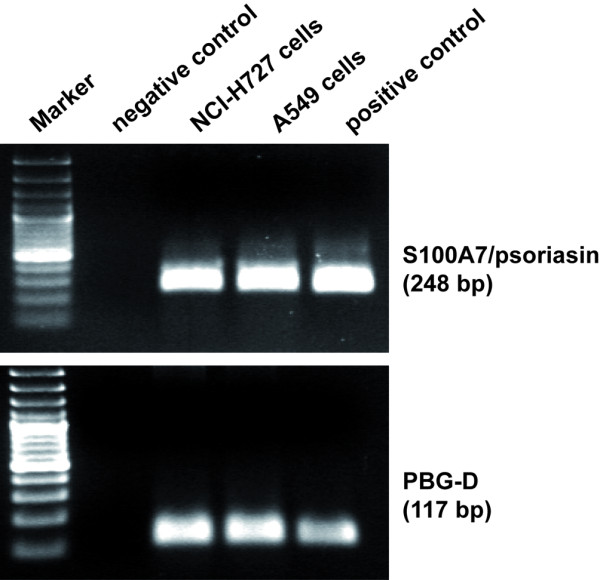
**Human bronchial and lung epithelial cells express S100A7/psoriasin mRNA**. RT-PCR was performed with specific primers for S100A7/psoriasin and PBG-D as an internal control on NCI-H727 and A549 cells. The expected size of PCR products is 248 bp for S100A7/psoriasin and 117 bp for PBG-D. PCR products were separated on 1.5% agarose gel containing ethidium bromide. PCR reaction in absence of cDNA was used as a negative control, plasmid DNA as a positive control, 100-bp ladder as Marker.

**Figure 2:**
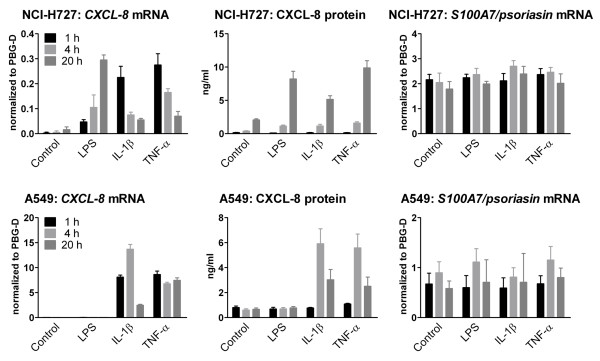
**S100A7/psoriasin mRNA levels remain unchanged after treatment with Toll-like receptor ligands or proinflammatory cytokines**. NCI-H727 (upper panel) and A549 cells (lower panel) were treated with 100 ng/ml LPS, 50 U/ml IL-1β, 5 ng/ml TNF-α, or medium alone (Control) for the time indicated. CXCL-8 mRNA (left) and S100A7/psoriasin mRNA (right) were analyzed by quantitative real-time PCR, and data are presented as mRNA level of both genes relative to PBG-D. CXCL-8 release (middle) was determined by ELISA. All values are depicted as mean ± SEM of three independent experiments.

S100A7/psoriasin has been shown to be the primary antimicrobial peptide of human skin for the killing of *E. coli *[14]. However, the expression of S100A7/psoriasin in the airways of human lungs has not been thoroughly investigated before. Thus, we first tried to identify the expression of S100A7/psoriasin mRNA in the human bronchial and lung epithelial cell lines NCI-H727 and A549, respectively. Figure 1 represents S100A7/psoriasin mRNA expression in both cell lines. Although S100A7/psoriasin is constitutively expressed at low levels, its expression can be further upregulated in keratinocytes upon challenging with proinflammatory cytokines such as IL-1 or TNF-α [14]. To further analyze whether lung epithelial cells could also be stimulated for the induction of an enhanced S100A7/psoriasin expression, we treated the cells with the TLR4 ligand LPS, as well as with the proinflammatory cytokines IL-1β and TNF-α but found that S100A7/psoriasin mRNA expression remained unchanged over at least 20 hours, regardless of the type of stimuli used (Figure 2, right panels). On the other hand, in NCI-H727 cells all three inflammatory stimuli induced increased CXCL-8 mRNA and protein expression (Figure 2, upper left and middle panels), albeit with a longer time course for the protein expression, while in A549 cells LPS, unlike the cytokines, was ineffective with respect to both mRNA and protein (Figure 2, lower left and middle panels). Showing the same results, whole bacteria preparations from different Gram-negative and Gram-positive bacteria have been used (data not shown).

**Figure 3:**
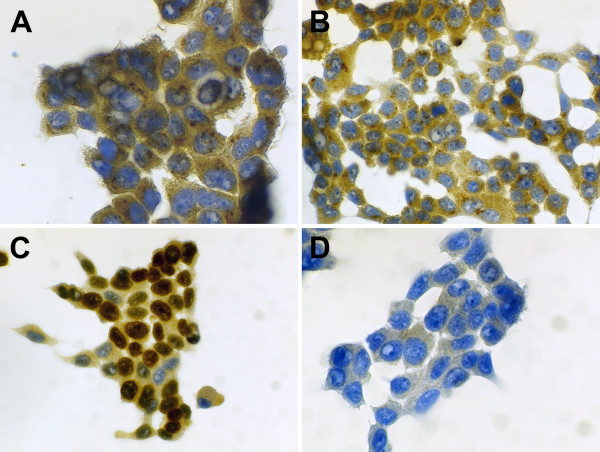
**S100A7/psoriasin is expressed in small perinuclear granules**. S100A7/psoriasin immunocytochemical detection was performed on (A) untreated and (B) LPS-treated (100 ng/ml, 21 hrs) HOPE-fixed NCI-H727 cells using the monoclonal Psoriasin/HID5/S100A7 (Imgenex, clone 47C1068) antibody. (C) Positive control is shown by the expression of the nuclear Ki67 antigen with the MIB-1 (2 μg/ml) antibody. (D) Negative control was included omitting the primary antibody. The results shown are representative of at least three independent experiments. Magnification: 600× (A), 400× (B, C, D)

**Figure 4:**
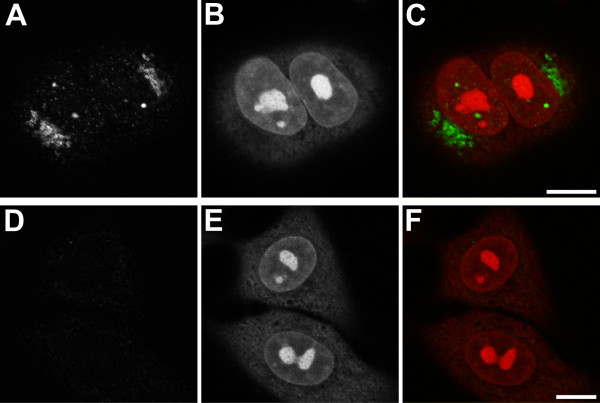
**S100A7/psoriasin exhibits both, nuclear and perinuclear localization**. (A) Endogenous S100A/psoriasin was visualized in A549 cells by confocal microscopy using the monoclonal Psoriasin/HID5/S100A7 antibody (Imgenex, clone 47C1068). (B and E) Nuclei were visualized with Toto-3 iodide (Molecular Probes). (D) Negative control was included by using mouse immunoglobulin isotype control antibody (IgG1). (C and F) Overlap between the S100A7/psoriasin staining or control IgG1 staining and nuclei staining, respectively, are shown in the merge panels. Bars: 10 μm. The results shown are representative of at least three independent experiments.

Since the detection of S100A7/psoriasin mRNA in bronchial and lung epithelial cells may not necessarily reflect the protein expression of S100A7/psoriasin, we used two different approaches to show S100A7/psoriasin protein expression in these cells. First, using immunocytochemistry, we analyzed protein expression in NCI-H727 cells (Figure 3). Again, we additionally investigated if stimulation of the cells with the TLR4 ligand LPS would alter the S100A7/psoriasin expression. The expression of the nuclear Ki67 antigen with the MIB-1 antibody serves as a positive control (Figure 3C). The results clearly show S100A7/psoriasin protein expression in NCI-H727 cells, which appears to be localized in small perinuclear granules (Figure 3A and 3B). However, as already seen with S100A7/psoriasin mRNA, the expression level remained unchanged after stimulation (Figure 3B). Secondly, to investigate S100A7/psoriasin protein expression in A549 cells, we used confocal microscopy. As can be seen in Figure 4, S100A7/psoriasin is clearly expressed. Similar to the results obtained with NCI-H727 cells, S100A7/psoriasin expression is strongly concentrated in perinuclear granules (Figure 4A-C). In addition, a focal nuclear expression could also be detected.

**Figure 5:**
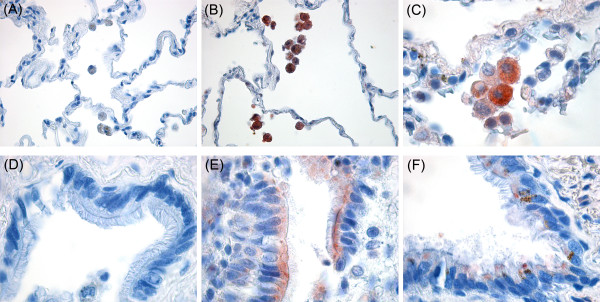
**S100A7/psoriasin is expressed in lung epithelial cells and alveolar macrophages**. S100A7/psoriasin immunohistochemical detection was performed on HOPE-fixed, paraffin-embedded lung tissue specimens using the monoclonal Psoriasin/HID5/S100A7 antibody (Imgenex, clone 47C1068). (A and D) negative controls without the primary antibody, (B and C) S100A7/psoriasin staining in alveolar macrophages, (E and F) S100A7/psoriasin staining in lung epithelial cells. Magnification: 100× (A, B); 400× (C-F). Results shown are representative of at least three independent experiments.

**Figure 6:**
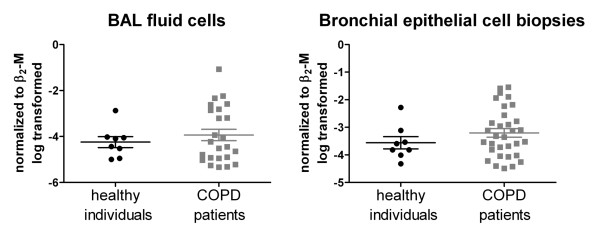
**S100A7/psoriasin mRNA expression remains unchanged during pathogenesis of COPD**. BAL fluid cells (left) and bronchial biopsies (right) were analyzed from COPD patients (N = 24 for BAL/N = 32 for biopsies) and healthy individuals (N = 8 in both). S100A7/psoriasin mRNA expression was measured by quantitative real-time PCR. Expression levels were normalized to the expression of β_2_-M mRNA and further log transformed. Results are depicted as mean ± SEM, each symbol representing a single probe.

After the clear evidence that S100A7/psoriasin is expressed in bronchial and lung epithelial cells we set out to investigate if its expression could also be detected in human lung tissue specimens (Figure 5). Indeed, S100A7/psoriasin could be found in alveolar macrophages (Figure 5B and 5C) as well as in lung epithelial cells (Figure 5E and 6F), as seen by immunohistochemistry in HOPE-fixed, paraffin embedded lung tissues.

Since S100A7/psoriasin is prominently involved in the antibacterial immune response of the human skin, we hypothesized that S100A7/psoriasin might also play a role in inflammatory responses of the lung in patients with COPD. Comparison of the expression levels of S100A7/psoriasin mRNA in BAL fluid cells and biopsy samples obtained from healthy individuals to that of patients with stage 1-4 COPD by quantitative real-time PCR did not reveal any significant differences (Figure 6). Variation of S100A7/psoriasin mRNA expression was slightly higher in the COPD group in both BAL fluid cells and biopsy samples. In addition, there was also neither a significant difference of S100A7/psoriasin mRNA expression with respect to disease stages (s. Additional file 1a) nor did we detect any correlation of S100A7/psoriasin mRNA expression with FEV_1_, VC or FEV_1_/VC (s. Additional file 1b). However, the analysis of the correlation of S100A7/psoriasin mRNA expression with cigarette smoking (i.e., pack years; irrespective of health/COPD status) revealed a significant correlation in biopsies (Spearman r = 0.3266, P value = 0.026) but not in BAL fluid cells (Spearman r = 0.2934, P value = 0.0649, s. also Additional file 1c).

**Figure 7:**
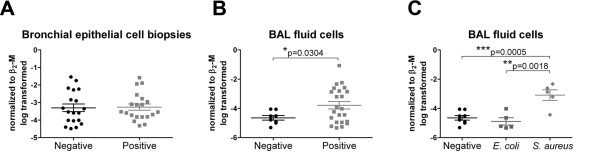
**S100A7/psoriasin mRNA expression is elevated upon pathogen exposure in BAL fluid cells**. S100A7/psoriasin mRNA expression level was compared on the basis of pathogen detection and independent of the COPD status. S100A7/psoriasin mRNA expression (A) in bronchial biopsies without (negative, N = 19) or with detection of pathogens (positive, N = 20); (B) in BAL fluid cells without (negative, N = 8) and with detection of pathogens (positive, N = 23); (C) in BAL fluid cells negative for a pathogen (N = 8) and positive for *E. coli *(N = 5) and *S. aureus *(N = 5). Differences between the groups were analyzed by unpaired *t*-test from log transformed data with p < 0.05 considered as significant.

All samples were additionally screened for detection of microbes. When we compared the S100A7/psoriasin mRNA expression in bronchial biopsies on the basis of bacterial detection and regardless of their COPD status, no differences could be seen (Figure 7A). However, in BAL fluid cells a significant increased S100A7/psoriasin mRNA level (p = 0.0304) could be found when bacteria had been detected (Figure 7B). However, this mRNA level in the bacteria positive group (positive) showed very high variation, ranging from an expression level seen in the pathogen negative group (negative) to up to 100 - 1000 fold higher expression. Since many different bacterial species were detected (s. Additional file 2 for a complete table), we could only form three groups for statistical analysis: negative BAL fluid cells without any bacteria detected and two groups, where we found either *E. coli *or *S. aureus*. Whereas BAL fluid cells positive for *E. coli *displayed a similar S100A7/psoriasin mRNA expression level as the control group, BAL fluid cells positive for *S. aureus *exhibited a significantly higher level of mRNA compared to the control group (p = 0.0005) and the *E. coli *group (p = 0.0018) (Figure 7C).

## Discussion

S100A7/psoriasin has been first identified and isolated from lesional psoriatic skin [28] and has been shown to act as a chemokine for T-cells and polymorphonuclear granulocytes [29]. S100 proteins have been implicated in innate immunity [30] and recently, S100A7/psoriasin has been shown to be the most important factor produced by immune system for the killing of *E. coli *on human skin [14]. Among the plethora of antimicrobial peptides, defensins and cathelicidins in particular have been shown to be expressed in the human lung (for review see [13]). However, the expression of S100A7/psoriasin in the human lung has not been thoroughly investigated.

Recently, Bryborn et al. could show that S100A7/psoriasin is one of several proteins found to be down-regulated in nasal lavage fluid of allergic patients [31] or in infected tonsils as well as in tonsils from allergic individuals [32]. This connection with allergy has been further substantiated with the association of a S100A7/psoriasin gene polymorphism with allergic rhinitis [33]. S100A7/psoriasin has also been found in whole saliva of patients with systemic sclerosis and lung involvement [34]. However, a study by Zhang et al., investigating the expression of S100A7/psoriasin in certain lung cancers, did not find any substantial protein expression in normal lung tissue [35] and the recent finding that S100A7/psoriasin is highly expressed in the tongue may have also been the reason for its detection in whole saliva [36]. In the present study, we clearly demonstrate that S100A7/psoriasin is expressed in human bronchial and lung epithelial cells and cell lines as well as in alveolar macrophages of healthy individuals and patients with COPD at different stages of the disease.

The expression of S100A7/psoriasin was upregulated upon stimulation of human keratinocytes with certain bacteria [37]. Recently, the ligand driving this upregulation has been identified as bacterial Flagellin [38]. After identifying the expression of S100A7/psoriasin mRNA and protein in the human lung epithelial cell lines we sought to determine if the level of expression depends on the activation status of the cells. We used typical activators of innate immune responses such as cytokines

(TNF-α or IL-1β) and whole bacteria (data not shown). However, in contrast to keratinocytes, the S100A7/psoriasin mRNA level did not change upon stimulation. The intracellular localization as well as the molecular mechanism of S100A7/psoriasin secretion has not been investigated so far. In both, bronchial and alveolar epithelial cells, S100A7/psoriasin protein was granular and expressed in confined perinuclear compartments. Interestingly, the amount of S100A7/psoriasin expressed appears to be rather high, indicating a potential prominent role of S100A7/psoriasin in the innate response of the bronchial and lung epithelium. This is further substantiated by the immunohistochemical protein staining of S100A7/psoriasin in human lung tissues, since it is particularly expressed in ciliated epithelial cells. In addition, we also show S100A7/psoriasin expression in alveolar macrophages.

In the present study we do not find any significant differences in the expression level of S100A7/psoriasin mRNA, when comparing COPD patients to healthy individuals, where disease in the COPD patients ranged from mild to very severe. This could indicate that S100A7/psoriasin plays only a minor role in chronic pulmonary inflammation in COPD. However, dividing all samples in groups depending on the status of pathogen detection (bacteria, fungi and/or virus), we identified a significant increase of S100A7/psoriasin mRNA in the group where microorganisms were detected in the lower respiratory tract. Although many different pathogens could be identified, most of the pathogens were only found once or twice, precluding any statistical analysis. Two bacterial strains, *E. coli *and *S. aureus*, have been detected in five samples each. In contrast to previous observations on the specific antimicrobial effectivity of S100A7/psoriasin in the skin [14], we found that samples of patients where *S. aureus *had been detected displayed a significant higher level of S100A/psoriasin mRNA than patients where *E. coli *has been detected. Moreover, S100A7/psoriasin mRNA levels were not elevated in patients positive for *E. coli *in their respiratory tract compared to those where no pathogens were detected. As the bactericidal efficiency of S100A7/psoriasin against *S. aureus *is much lower than against *E. coli *[14], these results could indicate that the regular constitutive levels of S100A7/psoriasin expression are sufficient to contain a potential *E. coli *infection but are inadequate to combat *S. aureus*.

## Conclusions

In summary, we found that S100A7/psoriasin is constitutively expressed in the lower human airways. Depending on bacterial occurrence in general and *S. aureus *in particular, S100A7/psoriasin is found to be upregulated and thus enhances the epithelial barrier function of the lower respiratory tract upon microbial challenge.

## Competing interests

The authors declare that they have no competing interests.

## Authors' contributions

EA contributed to conception and design of the study, carried out the experiments, analysis and interpretations of data and drafted the manuscript. CL contributed to conception and design of the study and acquisition of bronchial biopsies and BAL fluids. DS and TG carried out the immunoassays and contributed to the interpretation of data. NF and HS carried out the detection of microbes in patient material contributed to the interpretation of data. DB contributed to the acquisition of specimens of lung tissues. HH conceived of the study and participated in its design and coordination and was involved in revising the manuscript critically for important intellectual content. All authors read and approved the final manuscript.

## Pre-publication history

The pre-publication history for this paper can be accessed here:

http://www.biomedcentral.com/1471-2466/11/10/prepub

## Supplementary Material

Additional file 1 **S100A7/psoriasin mRNA expression in different COPD stages and correlation with lung function parameters and cigarette smoking.** a) S100A7/psoriasin mRNA expression in healthy controls and COPD I-II/COPD III-IV disease stages. b) Correlation analysis of S100A7/psoriasin mRNA expression with FEV1, VC, and FEV1/VC. c) Correlation analysis of S100A7/psoriasin mRNA expression with cigarette smoking (pack years). Click here for file

Additional file 2 **• Bacteria detected in BAL or bronchial biopsies**. Click here for file
