# Supplementary material for: S100A7/psoriasin expression in the human lung: unchanged in patients with COPD, but upregulated upon positive S. aureus detection
Source: BMC Pulm Med. 2011 Feb 15;11:10. doi: 10.1186/1471-2466-11-10 (PMC3050873; doi:10.1186/1471-2466-11-10)
Supplement: Additional file 1 — S100A7/psoriasin mRNA expression in different COPD stages and correlation with lung function parameters and cigarette smoking. a) S100A7/psoriasin mRNA expression in healthy controls and COPD I-II/COPD III-IV disease stages. b) Correlation analysis of S100A7/psoriasin mRNA expression with FEV1, VC, and FEV1/VC. c) Correlation analysis of S100A7/psoriasin mRNA expression with cigarette smoking (pack years). [file 1471-2466-11-10-S1.PDF]

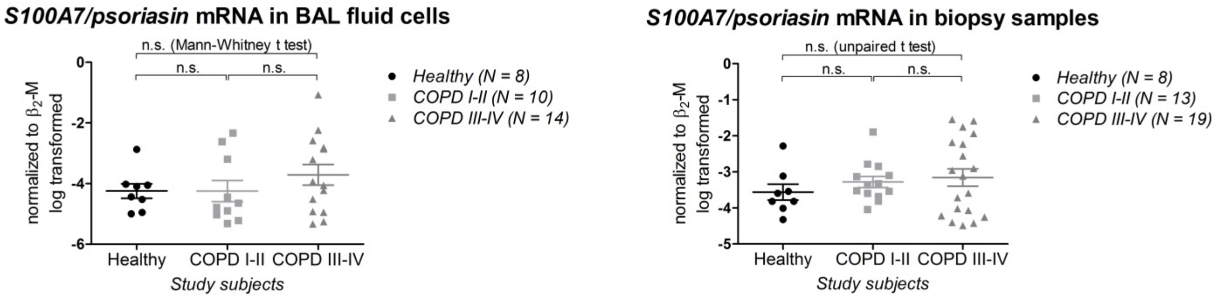

Additional file 1a. S100A7/psoriasin mRNA expression in healthy controls and COPD I-II / COPD III-IV disease stages. BAL fluid cells (left) and bronchial biopsies (right) were analyzed from COPD patients and healthy individuals (number in each group indicated). S100A7/psoriasin mRNA expression was measured by quantitative real-time PCR. Expression levels were normalized to the expression of β2-M mRNA and further log transformed. Results are depicted as mean ± SEM, each symbol representing a single probe.

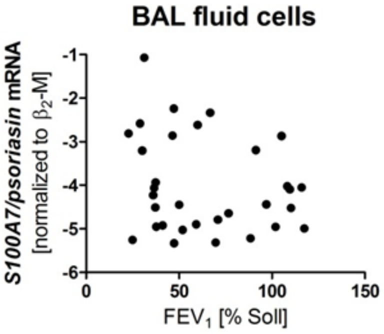

Number of XY Pairs	32
Spearman r	-0.2075
95% confidence interval	-0.5265 to 0.1628
P value (one-tailed)	0.1273
P value summary	ns
Exact or approximate P value?	Gaussian Approximation
Is the correlation significant? (alpha=0.05)	No

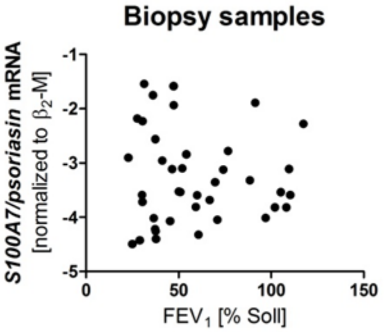

Number of XY Pairs	40
Spearman r	0.01295
95% confidence interval	-0.3085 to 0.3317
P value (one-tailed)	0.4684
P value summary	ns
Exact or approximate P value?	Gaussian Approximation
Is the correlation significant? (alpha=0.05)	No

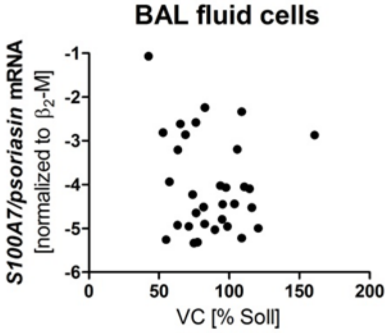

Number of XY Pairs	32
Spearman r	-0.1151
95% confidence interval	-0.4546 to 0.2535
P value (one-tailed)	0.2652
P value summary	ns
Exact or approximate P value?	Gaussian Approximation
Is the correlation significant? (alpha=0.05)	No

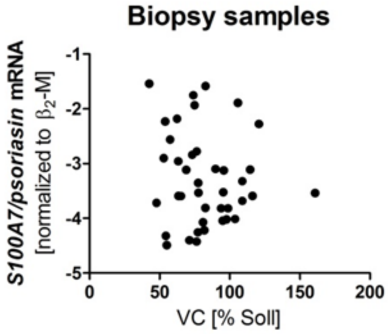

Number of XY Pairs	40
Spearman r	-0.09973
95% confidence interval	-0.4069 to 0.2277
P value (one-tailed)	0.2702
P value summary	ns
Exact or approximate P value?	Gaussian Approximation
Is the correlation significant? (alpha=0.05)	No

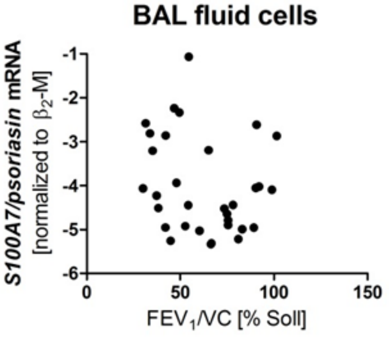

Number of XY Pairs	32
Spearman r	-0.2073
95% confidence interval	-0.5264 to 0.1630
P value (one-tailed)	0.1275
P value summary	ns
Exact or approximate P value?	Gaussian Approximation
Is the correlation significant? (alpha=0.05)	No

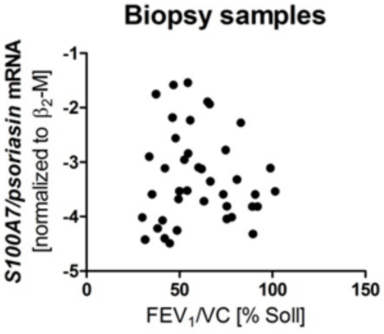

Number of XY Pairs	40
Spearman r	0.04184
95% confidence interval	-0.2821 to 0.3572
P value (one-tailed)	0.3988
P value summary	ns
Exact or approximate P value?	Gaussian Approximation
Is the correlation significant? (alpha=0.05)	No

Additional file 1b. Correlation analysis of S100A7/psoriasin mRNA expression with FEV_1, VC, and FEV_1/VC. Analysis of correlations between levels of S100A7/psoriasin mRNA and FEV_1,VC and the ratio FEV_1/VC in BAL fluid cells (left) and bronchial biopsies (right) was performed using Pearson correlations. Spearman r and P values are indicated below each graph. Each symbol represents a single sample.

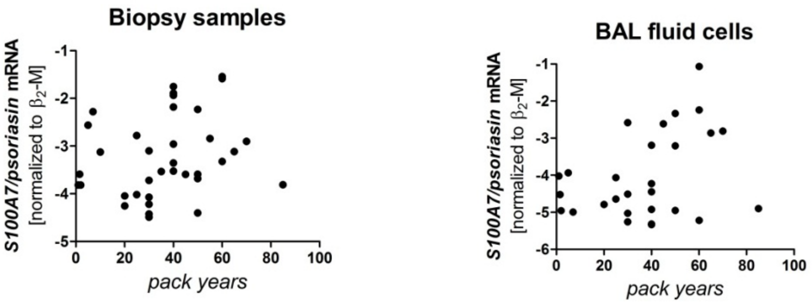

Number of XY Pairs	36
Spearman r	0.3266
95% confidence interval	-0.01239 to 0.5982
P value (one-tailed)	0.0260
P value summary	*
Exact or approximate P value?	Gaussian Approximation
Is the correlation significant? (alpha=0.05)	Yes

Number of XY Pairs	28
Spearman r	0.2934
95% confidence interval	-0.1011 to 0.6081
P value (one-tailed)	0.0649
P value summary	ns
Exact or approximate P value?	Gaussian Approximation
Is the correlation significant? (alpha=0.05)	No

Additional file 1c. Correlation analysis of S100A7/psoriasin mRNA expression with cigarette smoking (pack years). Analysis of correlations between levels of S100A7/psoriasin mRNA and pack years in BAL fluid cells (left) and bronchial biopsies (right) was performed using Pearson correlations. Spearman r and P values are indicated below each graph. Each symbol represents a single sample.
