# Supplementary material for: S100A7/psoriasin expression in the human lung: unchanged in patients with COPD, but upregulated upon positive S. aureus detection
Source: BMC Pulm Med. 2011 Feb 15;11:10. doi: 10.1186/1471-2466-11-10 (PMC3050873; doi:10.1186/1471-2466-11-10)
Supplement: Additional file 2 — • Bacteria detected in BAL or bronchial biopsies. [file 1471-2466-11-10-S2.PDF]

Additional file 2. Bacteria detected in BAL or bronchial biopsies

**Bacteria detected in BAL fluid**					
**status**	**Healthy**	**COPD I**	**COPD II**	**COPD III**	**COPD IV**
**# of samples**	9	2	10	14	3
**antibiotic use**			Avalox (1), Tavanic (1)	Augmentan (1), Avalox (2), Klacid (2)	
**bacteria**	*Escherichia coli*	*Moraxella catarrhalis*	*Klebsiella oxytoca*	*Enterobacter cloacae*	*Escherichia coli*
	Staphylococcus aureus (2)	*Escherichia coli*	*Staphylococcus aureus (3)*	*Haemophilus influenza (2)*	*Prevotella gingivalis*
	Serratia marcescens		*Pseudomonas aeruginosa*	*Pseudomonas aeruginosa*	
	Streptococcus pneumoniae		*Escherichia coli (3)*	*Staphylococcus aureus*	
			Streptococcus pneumoniae (2)	*Hafnia alvei*	
			Enterococcus faecium	*Escherichia coli*	
				Stenotrophomonas maltophilia	
				Moraxella catarrhalis	
				Pseudomonas putida	

**Bacteria detected in bronchial biopsies**					
**status**	**Healthy**	**COPD I**	**COPD II**	**COPD III**	**COPD IV**
**# of samples**	10	2	12	15	4
**antibiotic use**			Avalox (1), Tavanic (1)	Augmentan (1), Avalox (2), Klacid (2)	
**bacteria**	*Staphylococcus aureus*		*Streptococcus pneumoniae (2)*	*Enterobacter cloacae*	
	Escherichia coli		*Staphylococcus epidermidis (2)*	*Enterococcus faecium*	
	Serratia marcescens		*Staphylococcus aureus (2)*	*Escherichia coli (2)*	
			Staphylococcus aureus MRSA	*Staphylococcus aureus*	
				Acinetobacter baumannii	
				Haemophilus influenza	
				Staphylococcus pasteuri	
				Pseudomonas aeruginosa	

Bacteria detected in BAL or bronchial biopsies in healthy controls or COPD disease stages. Number of samples per group obtained for analysis is indicated. Antibiotic use: Number in parenthesis indicates frequency per group.
